# Reduced expression of circRNA hsa_circ_0067582 in human gastric cancer and its potential diagnostic values

**DOI:** 10.1002/jcla.23080

**Published:** 2019-11-12

**Authors:** Xiuchong Yu, Haixiang Ding, Liangwei Yang, Yu Yu, Jiaming Zhou, Zhilong Yan, Junming Guo

**Affiliations:** ^1^ Department of Gastrointestinal Surgery Ningbo First Hospital Ningbo China; ^2^ Department of Biochemistry and Molecular Biology, and Zhejiang Key Laboratory of Pathophysiology Medical School of Ningbo University Ningbo China

**Keywords:** clinical significance, diagnostics, gastric cancer, hsa_circ_0067582, qRT‐PCR

## Abstract

**Background:**

Gastric cancer (GC) is one of the global mortality diseases and has a poor prognosis due to the lack of ideal tumor biomarkers. Circular RNAs (circRNAs) are an abundant kind of endogenous RNAs that recently are found play a crucial role in the cancer occurrence and development. Nevertheless, little is known with regard to the diagnostic values of these circRNAs for GC. In this article of research, we investigated the role of hsa_circ_0067582 in clinical diagnosis of GC.

**Materials and Methods:**

We used divergent primers, and the expression levels of hsa_circ_0067582 in 93 fresh GC tissues and paired adjacent normal tissues from surgical patients were detected using quantitative reverse transcription‐polymerase chain reaction (qRT‐PCR). Then, a receiver operating characteristic (ROC) curve was established to assess the diagnostic significance of hsa_circ_0067582. The relationship between expression of hsa_circ_0067582 and clinicopathological factors of patients was made further explored.

**Results:**

Hsa_circ_0067582 levels were significantly decreased in GC tissues contrasted with adjacent normal tissues (n = 93, *P* *<* .001). After that, we discovered that it was evidently downregulated in 81.7% (76/93) GC tissues. The area under the ROC curve (AUC) of hsa_circ_0067582 was up to 0.6937, the sensitivity was 66.67%, and the specificity was 61.29%. Moreover, the hsa_circ_0067582 levels were obviously associated with tumor diameter (*P* = .002) and carbohydrate antigen 19‐9 (CA19‐9, *P* = .01). Meanwhile, after operation, low‐level group of hsa_circ_0067582 of GC patients was associated with better prognosis.

**Conclusion:**

Our data imply that hsa_circ_000067582 may be a potential biomarker for GC diagnosis and prognosis evaluation.

## 1 INTRODUCTION

Gastric cancer (GC) is the fourth global malignant tumors and the fourth leading cause of cancer‐related death worldwide, especially in Eastern Asian countries.^1^ Though the overall survival of GC patients to some extent has improved due to the application of early detection technology and radical surgery, most early‐stage gastric cancer (EGC) patients have no characteristic manifestations, and many patients are still diagnosed at the advanced stages and miss the best chance to therapy.^2^ Nowadays, upper gastrointestinal (GI) endoscopy followed by pathologic examination remains the golden standard for the diagnosis of GC.^3^ However, upper GI endoscopy is not a routine medical examination in most countries, and making a gastroscopy inspection is an invasive technique that will sometimes make patients uncomfortable.^4^ On a global scale, a recent study showed that the 5‐year survival rate of stage I patients can be more than 90%, while that of the stage IV patients who already show distant metastases is only approximately 18%.^5^, ^6^ Yet, traditional serum tumor biomarkers such as carcinoembryonic antigen (CEA), carbohydrate antigen 19‐9(CA19‐9), and carbohydrate antigen 72‐4 (CA72‐4) are not very effective in the diagnosis of GC.^7^, ^8^ Their sensitivity and specificity are relatively low.^9^ Therefore, it is necessary to search for stable, non‐invasive, and cost‐effective biomarkers to improve the diagnosis and monitoring patients with GC.^10^

Circular RNAs are a particular kind of endogenous noncoding RNAs featuring closed ring structure and neither 5′ cap structure nor a 3′ polyadenylated tail.^11^ Thanks to the circular structure, circRNAs are not easy to be degraded by traditional RNA exonuclease and are more stable, conserved, and highly abundant than other linear noncoding RNAs.^12^, ^13^ Recent studies have demonstrated that circRNAs can be the potential novel biomarkers in diagnosing many tumors.^14^ Researchers identified that some candidate circRNAs can serve as biomarkers for the prostate cancer and were able to detect circRNAs in the urine.^15^ Another study revealed that oral squamous cell carcinoma (OSCC) tissues are rich in circRNAs and the results showed that hsa_circ_0008309 may be a potential biomarker for the OSCC.^16^ More importantly, circRNAs are considered as microRNA (miRNA) sponges and can influence the activity of some proteins.^17^ Besides, downregulation of circTRIM33‐12 acts as a sponge of microRNA‐191 to affect hepatocellular carcinoma (HCC) progression.^18^ Hsa_circ_0009361 acts as a sponge of miR‐582 to repress colorectal cancer progression.^19^ Due to these biological properties, circRNAs may be important biomarkers for the occurrence and development of cancer.^20^

With the development of high‐throughput sequencing technique and bioinformatic analysis, an increasing number of cancer‐related circRNAs have been discovered.^21^ In this article of study, we focused our attention on hsa_circ_0067582 (http://www.circbase.org/cgi-bin/singlerecord.cgi?xml:id=hsa_circ_0067582) based upon the results of our previous microarray analysis, and the expression of circRNAs in GC tissues is different from adjacent non‐tumorous tissues (GEO No.GSE89143, Guo, 2016: https://www.ncbi.nlm.nih.gov/geo/query/acc.cgi?acc=GSE89143). The expression of hsa_circ_0067582 was quite different in GC tissue compared to paired normal tissue. So, we chose hsa_circ_0067582 as a research target to analyze its diagnostic values in GC. The gene of hsa_circ_0067582 is located at chromosome 3:141231004‐141259451. Its spliced sequence length is 394 nt; the associated gene symbol is RAS p21 protein activator 2 (RASA2). To our knowledge, it is the first time in our study to identify the roles of hsa_circ_0067582. Here, qRT‐PCR was utilized to explore the expression of hsa_circ_0067582 in GC patients; the relationship with clinicopathological factors was analyzed as well. Our results advocated that hsa_circ_0067582 may play as a diagnostic biomarker in GC.

## 2 MATERIALS AND METHODS

### 2.1 Specimens and clinical information collection

We made a collection of clinical GC tissue samples from Yinzhou People's Hospital in Ningbo between January 2010 and December 2015. Before any treatment was applied, the 93 GC tissues and their paired adjacent non‐tumorous tissues were collected from surgical operations. Gastric cancer tissues were taken from the mucosa of the center of the tumor. The corresponding adjacent non‐tumorous tissues were obtained from the mucosa 5 cm beyond the edge of carcinoma, contained not any tumor cells evaluated by two well‐experienced pathologists. Fresh specimens were soaked in the RNA fixer Reagent (BioTeke) after being immediately removed from the patients' bodies, and then conserved at −80°C until we use.

The clinical information of all samples was collected, and the GC diagnosis was finally confirmed by histopathology. The pathologic stages of these neoplasms were determined based on the tumor node metastasis (TNM) staging system of the International Union Against Cancer (8th edition).^22^, ^23^ The histological grades were assessed according to the American Joint Committee on Cancer (AJCC) cancer staging manual (8th edition).^24^, ^25^ Furthermore, no chemotherapy, radiotherapy, or targeted therapy was used to these patients before the operation and gastroscopic biopsy.

All the patients had consented to take part in this study and provided written informed consent. The Human Research Ethics Committee of Ningbo University agreed with all parts of this research (IRB No. 20120303).

### 2.2 Total RNA extraction and quality control

Following the manufacturer's instructions, total RNA was got from the GC tissues and the paired adjacent non‐tumorous tissues using the reagent of TRIzol (Invitrogen). The purity and the concentration of total RNA were detected with the machine of DS‐11 Spectrophotometer (DeNovix). The A260/A280 ratio was used to evaluate the RNA purity, within 1.8‐2.0 for the samples that were qualified. Finally, the RNA was well preserved at refrigerator −80℃ until we use.

### 2.3 Reverse transcription

According to the manufacturer's instructions, total RNA was performed to synthesize cDNA using GoScript RT System (Promega) with random primers.

### 2.4 qRT‐PCR ulteriorly detection of hsa_circ_0067582

According to the manufacturer's instructions, the expression levels of hsa_circ_0067582 were analyzed by qRT‐PCR using GoTaq qPCR Master Mix (Promega) implemented on a Mx3005P real‐time PCR System (Stratagene). The sequences of divergent primers were first designed by Primer 3 and BLAST (NCBI) and then synthesized by BGI (Shenzhen) Co, Ltd. The divergent primer sequences well overlapped the splice junction used in the detection of hsa_circ_0067582 were 5′‐AGAAGACTTGTGTAATCACAG‐3′ and 5′‐ AATTTTTTGCTTCACTGTAC‐3′. The sequences of convergent primers (glyceraldehyde 3‐phosphate dehydrogenase (GAPDH), used to normalize the levels of the circRNA) were 5′‐CTGCCAACGTGTCAGTGGTG‐3′ and 5′‐TCAGTGTAGCCCAGGATGCC‐3′. The reaction conditions of thermal cycling were as follows: hot start at 95℃ for 5 minutes, next 40 cycles at 95℃ for 15 seconds, 56℃ for 30 seconds, and 72℃ for 30 seconds. The qRT‐PCR products were confirmed by Sanger sequencing, which was completed by Geneseed. The relative quantification levels of hsa_circ_0067582 were calculated using the ΔC*q* (quantification cycle) method.^26^ A higher ΔC*q* value indicated a lower expression level.^27^ Through three independent rigorous experiments, all of data were expressed by mean ± standard deviation (SD).

### 2.5 Statistical analysis

All the experimental data were analyzed by the Statistical Product and Service Solutions software 19.0 software (SPSS). Figures and tables were produced by GraphPad Prism 5.0 (GraphPad Software). The data in this article are in accordance with the normal distribution. The Shapiro‐Wilk test, Student's *t* test, one‐way analysis of variance (ANOVA), and Kruskal‐Wallis test were flexibly applied according to the actual conditions. A value of *P* < .05 (two‐sided) was considered as statistically significant.

## 3 RESULTS

### 3.1 Characterization and amplification of hsa_circ_0067582

**Figure 1:**
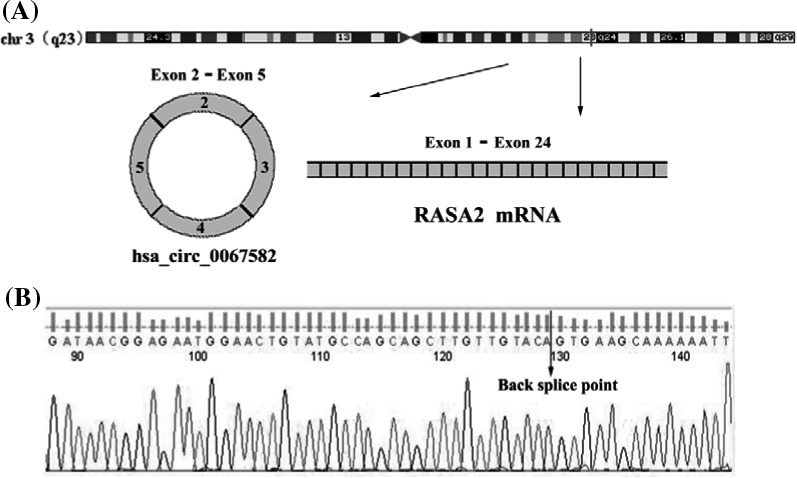
Characterization and amplification of hsa_circ_0067582. A, Hsa_circ_0067582 is encoded from chromosomal region 3q23. In this region, the typical transcript is RAS p21 protein activator 2 (RASA2) mRNA includes 24 exons. Four exons of them form hsa_circ_0067582 from exon 2 to exon 5. B, Sanger sequence results of qRT‐PCR products of hsa_circ_0067582 in GC tissues. qRT‐PCR: quantitative reverse transcription‐polymerase chain reaction

The hsa_circ_0067582 is encoded from chromosomal region 3q23. In this region, the typical transcript is RAS p21 protein activator 2 (RASA2) mRNA, which consists of 24 exons. Four exons of them formed hsa_circ_0067582, from exon 2 to exon 5 (Figure 1A). We designed one pair divergent primers according to the special circular structure to amplify the hsa_circ_0067582. To confirm the correction of qRT‐PCR, specificity of the hsa‐circ‐0067582 amplified products was analyzed. Firstly, our melting curve data showed that amplified product yielded only a single peak. The analysis signified that there was neither primer dimers nor non‐specific amplification. Secondly, we used Sanger sequencing and analyzed the amplified product. These results showed that the sequence of hsa_circ_0067582 PCR products contains the cyclization site (Figure 1B), and the sequences were completely coincident with that in circBase, the website of circBase (http://circbase.org/). The PCR product length was 168 nt. The data showed that hsa_circ_0067582 exists in the tissues of GC and could be amplified by qRT‐PCR availably.

### 3.2 Downregulated expression of hsa_circ_0067582 in GC patients

**Figure 2:**
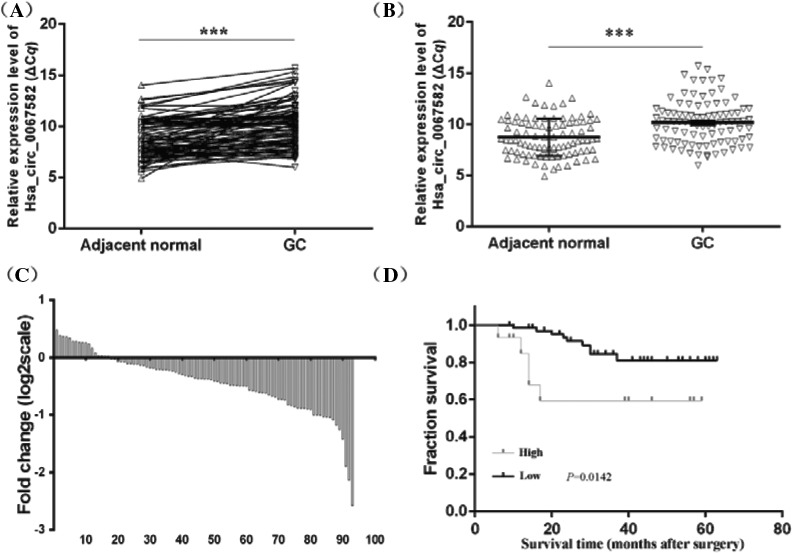
The expression levels of hsa_circ_0067582 in gastric cancer samples. Statistical significance was defined as two‐sided ***<0.001. A, Expression levels of hsa_circ_0067582 in each patient, with comparison between tumor tissues and the normal adjacent tissues (n = 93). Higher ΔC*q* value indicates lower expression. ***P < .001. B, The expression levels of hsa_circ_0067582 were significantly lower than those in adjacent normal tissues (n = 93, *P* < .001). C, The percentage of low expression of hsa_circ_0067582 in GC tissues accounts for 81.7% (76/93). D, Kaplan‐Meier analysis of OS based on hsa_circ_0067582 expression in all GC patients

We detected the expression levels of hsa_circ_0067582 in 93 GC tissues and the paired normal adjacent tissues using qRT‐PCR. The difference in ΔC*q* between cancer and adjacent normal tissues was in accordance with the normal distribution. Results showed that hsa_circ_0067582 was significantly lower in GC tissues than those in adjacent normal tissues (*P* < .001, Figure 2A,B). Among all samples, the lower expression samples accounted for more than 81.7% (76/93, Figure 2C). Next, hsa_circ_0067582 levels in tumor tissues were categorized as two groups, high group and low group, according to the 2^−ΔΔC^ *^q^* level. The results indicated that patients in the low hsa_circ_0067582 group had a much longer overall survival than those in high group (*P* = .0142; Figure 2D).

### 3.3 Hsa_circ_0067582 has potential diagnostic values in GC

As above results indicated, hsa_circ_0067582 showed markedly downregulated in GC tissues (Figure 2). We made further efforts to assess the connection between the hsa_circ_0067582 expression and the patients' clinicopathological features. As described in Table 1, the expression levels of hsa_circ_0067582 were closely associated with tumor diameter (*P* = .002) and CA19‐9 (*P* = .01). These clearly indicated that larger tumor diameter tumor patients were associated with lower expression levels of hsa_circ_0067582. These study results showed that CA19‐9‐positive patients had higher hsa_circ_0067582 levels. Besides, no relationship was detected between its level and the other clinicopathological factors.

**Table 1:** The relationship between hsa_circ_0067582 expression levels (ΔC*q*) in GC tissues and clinicopathological factors of GC patients

Characteristics	No. of cases (%)^a^	Mean ± SD	*P* value
Age(y)			
<60	23 (27.1)	10.54 ± 2.28	.726
≥60	62 (72.9)	10.36 ± 2.03	
Gender			
Male	64 (75.3)	10.28 ± 2.15	.326
Female	21 (24.7)	10.8 ± 1. 9	
Diameter (cm)			
<5	37 (43.5)	9.65 ± 1.89	**.002**
≥5	48 (56.5)	11 ± 2.06	
Differentiation			
Well	5 (5.9)	9.56 ± 2.57	.355
Moderate	19 (22.3)	10.93 ± 2.21	
Poor	61 (71.8)	10.32 ± 2.02	
Lymphatic metastasis			
N0	29 (34.1)	10.1 ± 2.35	.486
N1	16 (18.8)	10.85 ± 2.01	
N2	10(11.8)	11.06 ± 2.16	
N3	30 (35.3)	10.27 ± 1.86	
Invasion			
T1&TIS	15 (17.65)	10 ± 2.21	.723
T2	9 (10.59)	10.86 ± 1.4	
T3	7 (8.23)	10.01 ± 2.87	
T4	54(63.53)	10.5 ± 2.08	
Distal metastasis			
M0	77 (90.6)	10.37 ± 2.11	.571
M1	8 (9.41)	10.82 ± 1.98	
TNM stage			
I&II	37 (43.5)	10.48 ± 2.46	.787
III&IV	48 (56.5)	10.36 ± 1.79	
CEA			
Positive	75 (88.2)	10.38 ± 2.14	.723
Negative	10 (11.8)	10.64 ± 1.75	
CA19‐9			
Positive	47 (55.3)	9.89 ± 2.06	**.01**
Negative	38 (44.7)	11.06 ± 1.97	

Abbreviation: SD, standard deviation.

Tumor diameter (P=0.002) and carbohydrate antigen 19‐9 (CA19‐9, P=0.01). Bold values mean *P* <  .05.

a 8 Patients are not included due to incomplete clinicopathological information.

**Figure 3:**
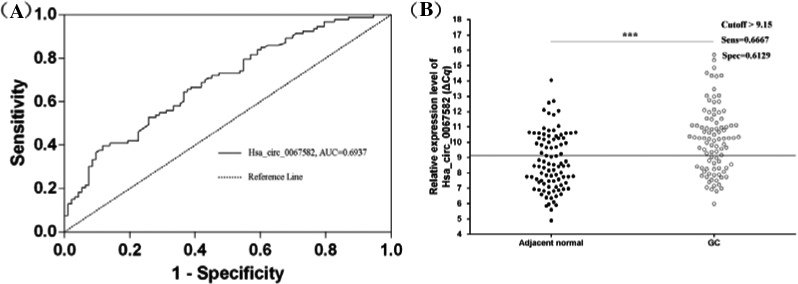
The diagnostic values of hsa_circ_0067582 in gastric cancer. A, The ROC curve of hsa_circ_0067582 in the differentiating GC tissues from controls. The area under the curve was up to 0.6937. B, The cut‐off value, sensitivity, and specificity were established by ROC curve. ROC, receiver operating characteristic

In order to have a better evaluate the diagnostic values, ROC curve was established to test the potential diagnostic value of hsa_circ_0067582 (Figure 3A,B). The area under the ROC curve (AUC) reached 0.6937, with 66.67% sensitivity and 61.29% specificity, respectively. The optimal cut‐off thresholds were defined by highest Youden index. The cut‐off value (ΔC*q*) was 9.15. Taken together, this research speculated that hsa_circ_0067582 might have potential values in detecting of GC.

### 3.4 The binding miRNAs of hsa_circ_0067582 predicted by bioinformatic analysis

Many circRNAs have complementary sites with miRNA‐teamed miRNA response elements (MREs). Given that miRNAs function as downstream regulatory elements of circRNAs to affect the progression of GC, identification of the signal axis from circRNAs to miRNAs is important. We predicted the potential binding miRNAs for hsa_circ_0067582 using bioinformatic analysis.

The results showed there were 18 potential binding miRNAs (Table S1). According to these predicted circRNA‐miRNA sponging sites, hsa_circ_0067582 may bind certain miRNAs, and their verified functions indicated a promising role involved in the adjustment of GC progression.

## 4 DISCUSSION

CircRNAs have two major advantages: remarkably stable and highly conserved. With these two important properties, circRNAs have certain advantages over other noncoding RNAs in the diagnosis and treatment of cancer.^28^ Though more and more academic scholars have started pay attention to, and begun to explore the potential roles of circRNAs, the clinical diagnostic values in tumor remain not well known.

The prognosis of GC is tightly associated with disease stage. Patients with later stages have lower 5‐year survival rate. So, establishment of an early cancer screening system is important.^29^ In recent years, a number of studies have focused on finding ideal biomarkers. CircRNAs are acting in the regulation of cancer progression.^30^ The goal of our study is to explore the relationship between circRNAs and GC. In our research, we for the first time found the expression levels of hsa_circ_0067582 were lower in GC tissues compared with adjacent normal tissues (Figure 2A,B). Sufficient tumor surgical resection is the key therapeutic factor for resectable GC because gastrectomy plus D2 lymphadenectomy has contributed to raise survival rates.^31^ Patients with low hsa_circ_0067582 had a much longer overall survival than those with high levels (Figure 2D). We hypothesized that the low hsa_circ_0067582 group had a better recovery after operation. This result showed that hsa_circ_0067582 may be a possible biomarker and a prognostic indicator contributes to GC screening.

The association between circRNAs and some clinicopathological features has strong clinical value.^26^ This research results showed the hsa_circ_0067582 levels in GC tissues were significantly related to some clinicopathological factors, such as tumor diameter and CA19‐9 (Table 1). The patients with lower hsa_circ_0067582 level have larger tumor diameter. Tumor size is an important factor that influences prognosis of patients with GC. As we know, CA19‐9 is a glycoprotein and a common serum biomarker of gastrointestinal tumors.^32^ This connection between hsa_circ_0067582 and CA19‐9 in GC offers a clue for the diagnosis of GC. As a GC tissue‐based biomarker, the area under the ROC curve (AUC) of hsa_circ_0067582 reached 0.6937. The sensitivity was 0.6667, and the specificity was 0.6129. Its false‐positive rate was 0.3871 and false‐negative rate was 0.3333. The positive predictive value (PPV) was 0.6327, and negative predictive value (NPV) was 0.6477.

At present, we knew little about the mechanisms underlying circRNAs on cancer occurrence. The most recent researches indicated that circRNAs regulate with MREs and act as “competing endogenous RNA (ceRNA)”.^17^ Due to the revolutionary breakthrough in RNA sequencing technique and biophysics techniques, we predicted 18 potential miRNA binding sites of hsa_circ_0067582 using a bioinformation database (Table S1). Based on the sponge theory, hsa_circ_0067582 may act as a ceRNA to bind these miRNAs, which may take part in the regulation of GC progression. However, these predictions have not yet been verified. Next, we need to carry out a corresponding experiment to explore whether hsa_circ_0067582 regulates the function of gastric cancer by binding these sites.

The limitation of this study is that it is still a relative small sample study. It would be better if is initially validated on a smaller patient cohort (Training set) and then on a larger patient cohort (Validation set). To sum up, our results indicate that hsa_circ_0067582 may play a role as a potential biomarker for the diagnosis of GC. Through further deeply identifying the values of circRNAs, we could enhance our comprehension of the mechanisms of circRNAs in the related tumors.
