# Supplementary material for: Reduced expression of circRNA hsa_circ_0067582 in human gastric cancer and its potential diagnostic values
Source: J Clin Lab Anal. 2019 Nov 12;34(3):e23080. doi: 10.1002/jcla.23080 (PMC7083425; doi:10.1002/jcla.23080)
Supplement: Supplementary file 1 [file JCLA-34-e23080-s001.doc]

Table S1. The miRNAs may bind with hsa_circ_0067582

| CircRNA Mirbase ID | | CircRNA (Top)– miRNA (Bottom) pairing | CircRNA Start | CircRNA End | |
| --- | --- | --- | --- | --- | --- |
| [hsa_circ_0067582](http://www.circbase.org/cgi-bin/singlerecord.cgi?id=hsa_circ_0067582) (5' ... 3') [hsa-miR-1225-5p](http://www.mirbase.org/cgi-bin/mirna_entry.pl?acc=hsa-miR-1225-5p) (3' ... 5') | | AGGAAGAAGUUUAUCGUACCCAA  |||||||  GGGGGGUGACCCGGCAUGGGUG | 91 | 98 |  |
| [hsa_circ_0067582](http://www.circbase.org/cgi-bin/singlerecord.cgi?id=hsa_circ_0067582) (5' ... 3') [hsa-miR-1243](http://www.mirbase.org/cgi-bin/mirna_entry.pl?acc=hsa-miR-1243) (3' ... 5') | | AGAUUCCAAGAACUUUCCAGUAU  ||||||  GUGAGGAUAUUAACUAGGUCAA | 163 | 169 | |
| [hsa_circ_0067582](http://www.circbase.org/cgi-bin/singlerecord.cgi?id=hsa_circ_0067582)(5' ... 3') [hsa-miR-1264](http://www.mirbase.org/cgi-bin/mirna_entry.pl?acc=hsa-miR-1264)(3' ... 5') | UAGCCAUCAAAAAAGAAGACUUG  ||||| |||||||  UUGUCCACGAGUUUA---UUCUGAAC | | 244 | 250 | |
| [hsa_circ_0067582](http://www.circbase.org/cgi-bin/singlerecord.cgi?id=hsa_circ_0067582)(5' ... 3') [hsa-miR-1289](http://www.mirbase.org/cgi-bin/mirna_entry.pl?acc=hsa-miR-1289)(3' ... 5') | | UCAUUACAGCCUGUUGACUCCAA  |||||| UUUUACGUCUAAGGACCUGAGGU | 297 | 303 | |
| [hsa_circ_0067582](http://www.circbase.org/cgi-bin/singlerecord.cgi?id=hsa_circ_0067582)(5' ... 3') [hsa-miR-129](http://www.mirbase.org/cgi-bin/mirna_entry.pl?acc=hsa-miR-1289)8(3' ... 5') | | CCUUGAAUUAAAACUGAAUGAAC  ||||||   AUGUAGACCUGUCGGCUUACUU | 344 | 350 | |
| [hsa_circ_0067582](http://www.circbase.org/cgi-bin/singlerecord.cgi?id=hsa_circ_0067582)(5' ... 3') [hsa-miR-183](http://www.mirbase.org/cgi-bin/mirna_entry.pl?acc=hsa-miR-183)(3' ... 5') | | AAGCAAAAAAUUUAUUGCCAUAU  ||||||   UCACUUAAGAUGGUCACGGUAU | 19 | 25 | |
| [hsa_circ_0067582](http://www.circbase.org/cgi-bin/singlerecord.cgi?id=hsa_circ_0067582)(5' ... 3') [hsa-miR-337-3p](http://www.mirbase.org/cgi-bin/mirna_entry.pl?acc=hsa-miR-337-3p)(3' ... 5') | | ACAAAGAGAUCUCCGUAUAGGAA  |||||||   CUUCUUUCCGUAGUAUAUCCUC | 218 | 225 | |
| [hsa_circ_0067582](http://www.circbase.org/cgi-bin/singlerecord.cgi?id=hsa_circ_0067582)(5' ... 3') [hsa-miR-384](http://www.mirbase.org/cgi-bin/mirna_entry.pl?acc=hsa-miR-384)(3' ... 5') | | AAAGAGAUCUCCGUAUAGGAAAA  ||||||   AUACUUGUUAAAGAUCCUUA | 220 | 226 | |
| [hsa_circ_0067582](http://www.circbase.org/cgi-bin/singlerecord.cgi?id=hsa_circ_0067582)(5' ... 3') [hsa-miR-421](http://www.mirbase.org/cgi-bin/mirna_entry.pl?acc=hsa-miR-421)(3' ... 5') | | GGUUUUCAUUACAGCCUGUUGAC  |||| |||||||   CGCGGGUUAAUUACAGACAACUA | 292 | 298 | |
| [hsa_circ_0067582](http://www.circbase.org/cgi-bin/singlerecord.cgi?id=hsa_circ_0067582)(5' ... 3') [hsa-miR-432](http://www.mirbase.org/cgi-bin/mirna_entry.pl?acc=hsa-miR-432)(3' ... 5') | | AUUUUACUUUGAGAU--UCCAAGAA  ||| ||||||   GGUGGGUUACUGGAUGAGGUUCU | 152 | 158 | |
| [hsa_circ_0067582](http://www.circbase.org/cgi-bin/singlerecord.cgi?id=hsa_circ_0067582)(5' ... 3') [hsa-miR-487a](http://www.mirbase.org/cgi-bin/mirna_entry.pl?acc=hsa-miR-487a)(3' ... 5') | | UUGUCUUUCUAUGUU----UAUGAUAA  |||| ||||||   UUGACCUACAGGGACAUACUAA | 186 | 192 | |
| [hsa_circ_0067582](http://www.circbase.org/cgi-bin/singlerecord.cgi?id=hsa_circ_0067582)(5' ... 3') [hsa-miR-513a-3p](http://www.mirbase.org/cgi-bin/mirna_entry.pl?acc=hsa-miR-513a-3p)(3' ... 5') | | NNNNNNGUGAAGCAAAAAAUUUAU  ||| ||||||  GGAAGAGUCUUUCCACUUUAAAU | 11 | 17 | |
| [hsa_circ_0067582](http://www.circbase.org/cgi-bin/singlerecord.cgi?id=hsa_circ_0067582)(5' ... 3') [hsa-miR-520g](http://www.mirbase.org/cgi-bin/mirna_entry.pl?acc=hsa-miR-520g)(3' ... 5') | | UCAGUGAAGAAUUUUACUUUGAG  ||| ||||||  UGUGAGAUUUCCCUUCGUGAAACA | 142 | 148 | |
| [hsa_circ_0067582](http://www.circbase.org/cgi-bin/singlerecord.cgi?id=hsa_circ_0067582)(5' ... 3') [hsa-miR-520h](http://www.mirbase.org/cgi-bin/mirna_entry.pl?acc=hsa-miR-520h)(3' ... 5') | | UCAGUGAAGAAUUUUACUUUGAG  ||| ||||||   UGAGAUUUCCCUUCGUGAAACA | 142 | 148 | |
| [hsa_circ_0067582](http://www.circbase.org/cgi-bin/singlerecord.cgi?id=hsa_circ_0067582)(5' ... 3') [hsa-miR-543](http://www.mirbase.org/cgi-bin/mirna_entry.pl?acc=hsa-miR-543)(3' ... 5') | | CUAUGUUUAUGAUAAGAAUGUUU  |||||||   UUCUUCACGUGGCGCUUACAAA | 194 | 200 | |
| [hsa_circ_0067582](http://www.circbase.org/cgi-bin/singlerecord.cgi?id=hsa_circ_0067582)(5' ... 3') [hsa-miR-570](http://www.mirbase.org/cgi-bin/mirna_entry.pl?acc=hsa-miR-570)(3' ... 5') | | UGGCAAAGAAACUUG--GUUUUCAU  |||||| ||||||   CGUUUCCAUUAACGACAAAAGC | 278 | 284 | |
| [hsa_circ_0067582](http://www.circbase.org/cgi-bin/singlerecord.cgi?id=hsa_circ_0067582)(5' ... 3') [hsa-miR-584](http://www.mirbase.org/cgi-bin/mirna_entry.pl?acc=hsa-miR-584)(3' ... 5') | | AGAGAUUGUUUCUGUACCAUAAA  ||||||   GAGUCAGGGUCCGUUUGGUAUU | 60 | 66 | |
| [hsa_circ_0067582](http://www.circbase.org/cgi-bin/singlerecord.cgi?id=hsa_circ_0067582)(5' ... 3') [hsa-miR-615-5p](http://www.mirbase.org/cgi-bin/mirna_entry.pl?acc=hsa-miR-615-5p)(3' ... 5') | | UUAUUGCCAUAUCUUGGACCCCA  |||||||   CUAGGCUCGUGGCCCCUGGGGG | 30 | 37 | |
| *CircInteractome*. Available: <https://circinteractome.nia.nih.gov/> | | | | | |
